# Sensing Acute Cellular Rejection in Liver Transplant Patients Using Liver-Derived Extracellular Particles: A Prospective, Observational Study

**DOI:** 10.3389/fimmu.2021.647900

**Published:** 2021-05-05

**Authors:** Kaan Kamali, Moritz Schmelzle, Can Kamali, Philipp Brunnbauer, Katrin Splith, Annekatrin Leder, Nadja Berndt, Karl-Herbert Hillebrandt, Nathanael Raschzok, Linda Feldbrügge, Matthäus Felsenstein, Joseph Gaßner, Paul Ritschl, Georg Lurje, Wenzel Schöning, Christian Benzing, Johann Pratschke, Felix Krenzien

**Affiliations:** ^1^ Department of Surgery, Charité – Universitätsmedizin, Freie Universität Berlin, Humboldt-Universität zu Berlin, Berlin Institute of Health, Berlin, Germany; ^2^ Berlin Institute of Health (BIH), Berlin, Germany

**Keywords:** acute cellular liver transplant rejection, extracellular particles (EP), FlowSOM, liver transplantation, liquid biopsy, t-SNE (t-Distributed Stochastic Neighbor Embedding), sensitivity and specificity

## Abstract

Acute cellular rejection (ACR) after liver transplantation (LT) goes along with allograft dysfunction, which is diagnosed by liver biopsy and concomitant histological analysis, representing the gold standard in clinical practice. Yet, liver biopsies are invasive, costly, time-intensive and require expert knowledge. Herein we present substantial evidence that blood plasma residing peripheral liver-derived extracellular particles (EP) could be employed to diagnose ACR non-invasively. *In vitro* experiments showed organ-specific EP release from primary human hepatocytes under immunological stress. Secondly, analysis of consecutive LT patients (n=11) revealed significant heightened EP concentrations days before ACR. By conducting a diagnostic accuracy study (n = 69, DRKS00011631), we explored the viability of using EP as a liquid biopsy for diagnosing ACR following LT. Consequently, novel EP populations in samples were identified using visualization of t-distributed stochastic neighbor embedding (viSNE) and self-organizing maps (FlowSOM) algorithms. As a result, the ASGR1^+^CD130^+^Annexin V^+^ EP subpopulation exhibited the highest accuracy for predicting ACR (area under the curve: 0.80, 95% confidence interval [CI], 0.70–0.90), with diagnostic sensitivity and specificity of 100% (95% CI, 81.67–100.0%) and 68.5% (95% CI, 55.3–79.3%), respectively. In summary, this new EP subpopulation presented the highest diagnostic accuracy for detecting ACR in LT patients.

## Introduction

Liver transplantation (LT) is the treatment of choice for patients with end-stage liver disease, acute liver failure, metabolic disorders, and selected liver tumors (1). Despite adequate postoperative care and immunosuppression, acute cellular rejection (ACR) frequently occurs in up to 40% of LT recipients, depending on the immunosuppressive regimen selected and the individual’s age (2, 3).

Early diagnosis and identification of ACR following LT is crucial for decreasing complications and maintaining adequate liver function during postoperative management. A liver biopsy (LB) is performed if, based on clinical observations and laboratory tests, including elevated transaminases and cholestasis parameters, ACR is suspected. Indeed, LB remains the current gold standard tool for securing ACR diagnosis, where histological features are analyzed (4). Every LB presents the risk of severe complications such as graft damage, infection, bleeding, and thrombosis, which is why LBs are controversial in terms of monitoring graft function and remain inconvenient due to the considerable time required and the need for a specialist to perform the biopsy (5). Besides, their diagnostic potential is heavily constrained by the high inter-observer variation among pathologists in the grading and the quality of the specimen. In this context, non-invasive and accurate monitoring tools can play an essential role in diagnosing ACR and individual postoperative care adaptation.

The hypothesized diagnostic utility of EP is based on the premise that the origin of the respective organ can be identified by examining the surface antigens. This would allow one to draw possible conclusions about organ function or dysfunction. We have previously shown that CD4^+^, CD8^+^ and CD31^+^ EP elevated in patients at risk for ACR (6). This exploratory study demonstrated EP as a risk predictor days before the ACR. Still, it did not test EP as a diagnostic biomarker, where index testing and reference testing were linked by time (4).

One of the prevalent cellular proteins used for detecting EP is annexin V (AnnV) (7, 8). Cells exposed to activation or apoptosis externalize phosphatidylserine (PS), which causes the corresponding cell to release EP after an increase in intracellular calcium concentration and cytoskeletal rearrangement (9). Due to its ability to bind to PS, which is primarily located in the cytosol facing side of the cell membrane, AnnV is a recognized cell membrane protein for identifying EP derived from different cell types. To identify liver originated EP, liver-specific antigens are required. The hepatocyte domain-specific plasma membrane protein, namely asialoglycoprotein receptor (ASGR1), is a C-type lectin receptor mostly expressed on mammalian hepatocytes responsible for binding, internalizing, and clearing glycoproteins (gp) containing asialoglycoproteins (10). Interestingly, the expression levels and localization of ASGR1 on hepatocytes change during liver inflammation, and ASGR1 overexpression has been described in HepG2 cells treated with interleukin (IL)-1, IL-6, or tumor necrosis factor (TNF-α) (11). As a floppase, MDR3 (multidrug resistance protein 3) P-gp is localized in the apical membrane of hepatocytes and transports phosphatidylcholine (12). Phosphatidylcholine release by ABCB4 is stimulated by canalicular bile salts (exported from ABCB11), increasing cholesterol export by ABCG5–ABCG8 (13). These two liver-specific transmembrane proteins potentially located on EP could be released during liver-specific stress but have not been investigated for ACR after LT thus far.

The immunological molecules that are involved in regulating ACR are gp CD130 and connexin 43 (Cx43). CD130 stabilizes the CD126–IL-6 complex and mediates signal transduction *via* IL-6 binding. This transmembrane domain subsequently induces the acute-phase response of hepatocytes (14) and therefore is a possible EP target during inflammation. Indeed, as a proinflammatory cytokine, IL-6 regulates T cell survival and differentiation, thereby critically affecting ACR in solid organ transplantation (15). Being a ubiquitous gap junction protein, Cx43 mediates communication between adjacent cells in liver tissue. Comprising the predominating gap junction type in the liver, Cx43 expression in hepatocytes and Kupffer cells is related to the severity of inflammation (16, 17).

Given this background, we investigated the role of liver-specific EP during ACR after LT. We conducted *in vitro* experiments to demonstrate EP’s release from primary hepatocytes under inflammatory stress as a proof-of-concept. In addition to the surface markers’ characterization, we investigated the EP’s application as a liquid biopsy for diagnosing ACR and performed a diagnostic accuracy study. In this setting, we used modern data visualization tools for identifying novel EP populations.

## Materials and Methods

### General Characteristics of the Study

This observational study complying with local regulatory guidelines and the Declaration of Helsinki was approved by the Charité ethics committee (Ethikkommission der Charité Universitätsmedizin Berlin) under vote number EA1/193/16 and was registered with the German Clinical Trials Register (DRKS00011631) and the World Health Organization (WHO) International Clinical Trials Registry Platform (18). Patients were enrolled consecutively at the Department of Surgery Charité from 12/09/2016 to 08/12/2017. The following inclusion criteria were used: ≥ age 18 years, male/female, liver transplantation, healthy control with no liver disease, written informed consent, ability to give information and consent. Patients were excluded when inclusion criteria were not met. A total of 69 patients were enrolled in the study, 50 of whom underwent LT and 19 were grouped as control patients. Note, STARD criteria for reporting diagnostic accuracy were considered for study design (**Supplementary Table 1**) (19).

### Surgical Procedure and Immunosuppression

All grafts were derived from brain-dead donors, and LT was performed as previously described (20). A standard regimen of immunosuppression consisted of tacrolimus (trough level week 1–4, 6–10 ng/mL; week 5–8, 5–8 ng/mL) or cyclosporin A (trough level week 1–4, 100–125 ng/mL; week 5–8, 75–100 ng/mL) and low-dose steroids. The steroids were subsequently tapered and discontinued completely three months after LT (from 40 mg to none). Mycophenolate mofetil was given additionally in patients with impaired renal function after transplantation. The primary cause of the liver disease was determined by histological examination of the explanted liver.

### Definition of Non-ACR and ACR Samples

Samples from the transplant patients were divided into histologically proven ACR and non-ACR subgroups. LB was only performed on clinical suspicion of graft rejection. The biopsy indication was based on laboratory tests, including elevated transaminases and cholestasis parameters. Senior pathologists evaluated the biopsies before EP analysis and graded rejection samples according to the Banff classification (21). Samples with histologically positive ACR findings were defined as biopsy-proven rejection samples.

### Definition of the Exploration and Control Group

The first 11 patients enrolled in the study who underwent LT were defined as “exploration group”, and their plasma was analyzed preoperatively and on POD (postoperative day) 1, 3, 7 and 14. Patients with symptomatic abdominal hernias were included in the control group. These patients had no history of diagnosed liver pathologies. Their blood samples were collected preoperatively and were analyzed as described below.

### Blood Sample Collection

Blood samples were obtained (blinded to the reference test) ≤3 days before to 2 days after a liver biopsy was performed or preoperatively (LT and control patients). Venous blood samples were drawn into 6 ml EDTA coated blood collection tubes according to the study protocol. The samples were centrifuged at 2500 ×*g* for 15 min at 4°C to separate the plasma from the corpuscular blood compartment. The isolated plasma was then split into 1-ml aliquots, which were promptly shock-frozen in liquid nitrogen and stored at -80°C.

### Liver Tissue Retrieval

A liver tissue sample was obtained from a patient without infectious diseases who had undergone partial hepatectomy. The tissue donor signed an informed consent form, and the project was approved by the local ethics committee (EA 1/289/16). The liver tissue was inspected macroscopically after the resection to locate the most physiologic site. The sample was carried in a transport unit filled with 4°C William’s E medium (Thermo Fisher Scientific, Gibco, Carlsbad, CA, USA, Cat. No. A1217601) under sterile conditions, keeping the cold ischemic time at a minimum.

### Hepatocyte Isolation and Culture

Primary hepatocytes were isolated from one donor liver tissue and cultured according to a protocol as previously described by our group (22). Briefly, the specimen was perfused with two solutions: one with EGTA and the other with collagenase P (Roche Diagnostics, Mannheim, Germany, Cat. No. 11 213857001). Afterwards, to avoid further digestion of hepatocytes, bovine serum albumin (Merck KGaA, Darmstadt, Germany, Cat. No. A3059-100G) was added to the cell suspension. The cell suspension was centrifuged to remove the cell debris. The hepatocytes were collected after centrifugation and resuspended in William’s E medium with supplements (1 µM Insulin [Lilly, Indianapolis], 1 µM Fortecortin [Merck Serono GmbH, Darmstadt, Germany], 1 mM sodium pyruvate, 10 mM HEPES-Buffer and 10% fetal calf serum [Biochrom AG, Berlin, Germany]), and then counted. The hepatocytes (1 million cells/well) were plated on 6 well collagen-coated plates; after 4 h, the dead cells were washed away, and the incubation process was started.

### Generation and Isolation of EP

The generation and isolation of S100-EP according to the protocol of Kornek et al. (23) was adopted previously by our group (24).

*In-vitro:* After incubation overnight, the hepatocytes were stimulated with concentrations 10 and 20 ng/ml of TNF-α to mimic inflammatory stress physiologically experienced in humans (25). One batch received no TNF-α as a negative control group. The supernatants (1 ml) of the wells were collected after TNF-α treatment, shock-frozen and stored at -80°C. Fluorescence-activated cell sorting (FACS) buffer (1x PBS, 1% BSA, 0.1% EDTA, 0.1% NaN3) was filtered with a 0.2 µm filter before use. Supernatants were thawed, and 800 µl of the supernatant was transferred to a new 1.5 ml Eppendorf tube. The tube was filled with 500 µl filtered FACS buffer and centrifuged at 10,000 ×g for 30 min at 4°C to remove remaining cell debris. Afterwards, 1300 µl supernatant was slowly transferred to a microcentrifuge polyallomer tube with a snap-on cap (Beckman Coulter, Brea, CA, USA, Cat. No. 357448) and centrifuged at 100,000 ×g for 95 min at 4°C in an ultracentrifuge (Beckman Coulter, Cat. No. A99833) to pellet the cell-derived biologically active S100 fraction (23). The pelleted EP were resuspended in 650 µl filtered FACS buffer, the EP suspension was split into 50 µl aliquots and stored at -80°C.

*Patient samples:* Fluorescence-activated cell sorting (FACS) buffer (1x PBS, 1% BSA, 0.1% EDTA, 0.1% NaN_3_) was filtered with a 0.2 µm filter before use. Plasma samples were thawed, and 800 µl plasma was transferred to a new 1.5 ml Eppendorf tube. The tube was filled with 500 µl filtered FACS buffer and centrifuged at 10,000 ×*g* for 30 min at 4°C to remove remaining platelets. Afterwards, 1300 µl supernatant was slowly transferred to a microcentrifuge polyallomer tube with a snap-on cap (Beckman Coulter, Brea, CA, USA, Cat. No. 357448) and centrifuged at 100,000 ×*g* for 95 min at 4°C in an ultracentrifuge (Beckman Coulter, Cat. No. A99833) to pellet the cell-derived biologically active S100 fraction (23). The pelleted EP were resuspended in 650µL filtered FACS buffer, the EP suspension was split into 50 µl aliquots and stored at -80°C.

Prior to staining, the aliquots were incubated overnight at 4°C with 0.2 µm filtered AnnV-binding buffer (BD Biosciences, Heidelberg, Germany, Cat. No. 556454).

### Labeling EP

Each sample containing 50 µl supernatant EP and 5 µL 10x AnnV binding buffer was subsequently incubated with antibodies: APC Alexa 700-conjugated Cx43 (clone: FAB7737N, R&D Systems, Minneapolis, MN, USA), BV421-conjugated CD130 (clone: AM64, BD Biosciences, San Jose, CA, USA), and PE-conjugated AnnV (Cat. No. 640908, BioLegend, San Diego, CA, USA), and FITC-conjugated ASGR1 (clone: REA608, Miltenyi Biotec, Auburn, CA, USA) or FITC-conjugated MDR3 (Cat. No. LS-C694886, LifeSpan BioSciences, Seattle, WA, USA) or FITC-conjugated CD31 (Cat. No. 303104, BioLegend). While in-vitro samples were labeled with APC Alexa 700-conjugated Cx43, BV421-conjugated CD130, PE-conjugated AnnV, and FITC-conjugated ASGR1, the patient samples were labeled additionally with FITC-conjugated MDR3 and FITC-conjugated CD31. The samples were incubated at room temperature for 15 minutes and then preserved on ice until measurement.

### Electron Microscopy

For the negative staining, carbon-coated mesh grids were hydrophilized with Alcian blue solution (1% in 1% acetic acid) followed by washing steps with distilled water. Next, 5µl of sample was placed on the grid and incubated for 10 minutes; the remaining liquid was removed through a filter paper after incubation time. After washing with distilled water, the grid was placed on a drop of freshly prepared 1% aqueous uranyl acetate solution for 20 seconds. Finally, the rest of the solution was removed by a filter paper and the grid was dried in a grid box. The imaging was performed on a Zeiss Leo 906 electron microscope at 80 kV acceleration voltage equipped with a slow scan 2K CCD camera (TRS, Moorenweis, Germany).

### EP Detection and Counting

**Figure 1:**
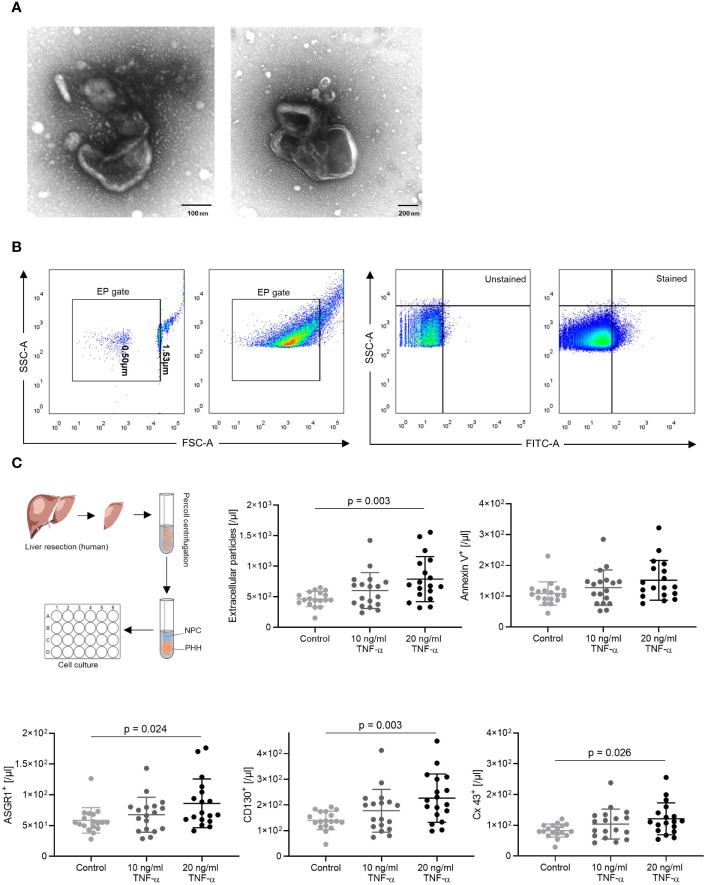
EP gating strategy and *in vitro* results. **(A)** Transmission electron microscopy images of EP. **(B)** The EP gate was defined using 0.5 µm and 1.53 µm calibration beads. EP population shift was observed after staining with ASGR1 antibodies. **(C)** *In vitro*, TNF-α induced human hepatocyte–derived EP. Primary human hepatocytes following liver resection were isolated, cultured overnight, and stimulated with 10 and 20 ng/ml TNF-α. EP surface antigens were stained, and absolute AnnV^+^, CD130^+^, Cx43^+^, ASGR1^+^ EP were analyzed. The ordinary one-way ANOVA with Tukey’s *post hoc* test was used. The plots are indicated by the mean, and all error bars indicate the SD.

Minimal Information for Studies of Extracellular Vesicles 2018 (MISEV2018) guidelines were adopted, and criteria regarding nomenclature, specimen collection and pre-processing, separation and concentration were applied, if applicable (26). The characterization and counting of S100-EP were achieved by flow cytometry as investigated by Kornek et al. (23). Our investigations under transmission electron microscopy showed a homogeneous structure surrounded by a double-layered electron-lucent cell membrane (**Figure 1A**). Prior to measurement, the flow cytometer was rinsed with FACS buffer that had been prefiltered through a 0.2 µm filter (Sartorius, Göttingen, Germany, Cat. No. ST16534-K). Filtered FACS buffer and single-antibody samples with FACS buffer were recorded to identify remaining background events. The EP were mixed evenly with 25 µL counting beads (Biolegend, San Diego, USA, Cat. No. 424902). For flow cytometric analysis, 0.8 µm deep-blue-dyed latex beads (Sigma, Cat. No. L1398) were first used for gating and voltage adjustment, as the beads are fluorescent and can be detected on forward and side scatter. For EP gating, silica particles (SiO_2_-R) in diameters of 0.501 µm and 1.53 µm (Microparticles GmbH, Berlin, Germany, Cat. No. SiO2-R-SC86 and SiO2-R-SC170-2, respectively) were used. The EP were quantified using counting beads and the following equation:

Equation 1 $$Absolute\;count\;(EP\;per\;µl\;plasma)=\frac{Corresponding\;positive\;events\times Beads\;volume\;x\;Beads\;concentration\;}{Bead\;events\;\times \;Aliquot\;volume}\times \;Dilution\;factor$$

The number of positive EP was calculated relative to the number of all gated EP. The flow cytometric analysis was performed on a FACS BD LSRFortessa™ flow cytometer (BD Biosciences, Heidelberg, Germany) and the data were analyzed using FlowJo software (Tree Star, Ashland, OR, USA).

### Data Visualization

For identifying novel EP populations and qualitative comparison between samples, we used viSNE (visualization of t-distributed stochastic neighbor embedding) and FlowSOM (self-organizing maps) algorithms. viSNE was executed using the default Cytobank (Santa Clara, CA, USA) parameters (iterations = 1000, perplexity = 30, θ = 0.5). For the analysis, internally compensated samples were used, and the flow cytometry data were transformed using hyperbolic arcsin with a cofactor of 150. A total of 241 319 selected events from three patient samples were randomly downsampled to 33 333 events per sample. All the figures generated were derived from the same viSNE run. viSNE maps were colored by channel to illustrate antigen expression. Based on antigen expression and FlowSOM algorithm, EP populations were clustered and displayed as viSNE maps. The number of expected metaclusters was set to 7.

### Statistics

The absolute and relative serum EP numbers were compared between ACR, non-ACR, and control samples. Data are presented as the mean with IQR and minima and maxima, if not stated in the figure legend. Categorical variables were compared using Fisher’s exact test. Continuous parameters such as laboratory results and absolute EP numbers and percentages were compared using the Mann-Whitney U test or the *t*-test for two groups. In comparison, data containing more than 2 groups were analyzed using one-way analysis of variance followed by Tukey’s *post hoc* test. In addition, a paired sample analysis was performed using the Wilcoxon matched-pairs signed ranked test. The area under the receiver operating characteristic curve (AUC) was generated to illustrate which EP subgroup demonstrated the highest diagnostic potential with respect to discriminating between ACR and non-ACR samples. The Youden index was calculated and used as a threshold for cut-off values. EP values when comparing ACR to non-ACR are shown by single data dots, and missing data were omitted when EP staining did not work. An overall alpha value of p < 0.05 was applied to reject the null hypothesis. GraphPad Prism 8.0 for Windows was used for statistical analysis (GraphPad Software, La Jolla, CA, USA).

## Results

### Release of EP by Primary Human Hepatocytes

To investigate the role of EP during ACR and prove whether hepatocytes release EP under inflammatory stress, we first isolated and cultivated primary human hepatocytes from patients who had undergone liver resection. The hepatocytes were cultured and subsequently stimulated with TNF-α (27–29), which induces T cell activation, the key reaction leading to allograft rejection. The EP were identified in FACS by forward and side scattering *via* calibration beads (**Figure 1B**).

We compared the control group with TNF-α treatment groups and detected a dose-dependent increase in EP [/μl] between the control and 20 ng/ml TNF-α treatment group (**Figure 1C**, p = 0.003). In addition, there was a significant increase in CD130^+^ (/µl) (p = 0.003), Cx43^+^ (/μl) (p = 0.026) and ASGR1^+^ (/µl) (p = 0.024) EP accompanied with an increasing trend of Ann V^+^ (/µl) EP. Interestingly, EP (%) showed no significant difference between groups when considering the relative ratios (**Supplementary Figure 1**). The findings show that, under TNF-alpha stimulation, mimicking an acute rejection-like microenvironment, human hepatocytes dose-dependently release EP.

### EP Dynamics Pre and Post LT

**Figure 2:**
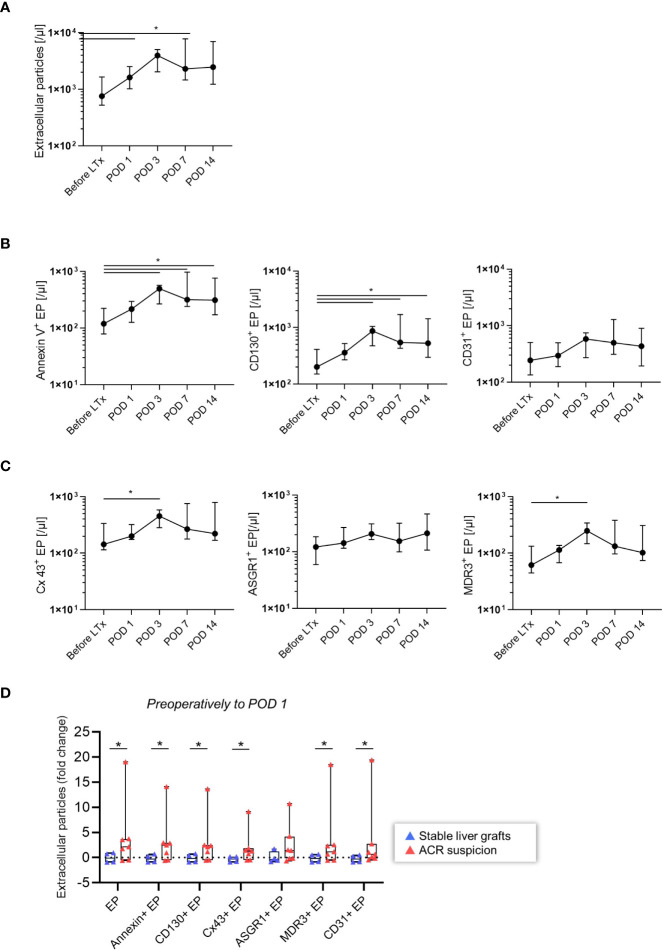
Time course of EP after LT (n = 11). **(A)** Total EP (/µl) **(B)** AnnV^+^, CD130^+^, CD31^+^ EP (/µl) **(C)** Cx43^+^, ASGR1^+^, MDR3^+^ EP (/µl) were stained and analyzed. The Wilcoxon matched-pairs signed ranked test was used. The plots are indicated by the median, and all error bars indicate the interquartile range (IQR). A single asterisk indicates significance at p < 0.05 **(D)** Patients at risk for acute rejection. EP dynamics from preoperative state to POD 1 of the total, AnnV^+^, CD130^+^, Cx43^+^, ASGR1^+^, MDR3^+^, CD31^+^ EP (/µl) and their fold change were analyzed. Absolute EP were non-normally distributed; the two-tailed Mann-Whitney U test was used. The plots are indicated by the mean, and all error bars indicate the SD.

Next, we explored the general dynamics of EP in patients who underwent LT. The demographic and clinical characteristics of the patient groups are presented in **Tables 1**, **2**. The EP were characterized based on liver-specific surface antigens (ASGR1, MDR3) and antigens that regulate immunological cascades (CD130, Cx43, CD31). We analyzed the isolated EP plasma samples of 11 patients before LT and on POD 1, 3, 7 and 14. In this time period, certain EP reached a peak at POD 3 (**Figure 2A**: total EP: p = 0.024; **B**: AnnV^+^ EP (/μl): p = 0.019, CD130^+^EP (/µl): p = 0.019; **C**: Cx43^+^ EP (/µl): p = 0.042 and MDR3^+^ EP (/µl): p = 0.019). After this upwards trend, a plateau phase ensued from POD 7 onwards. In contrast, ASGR1^+^ and CD31^+^ EP (/µl) did not show any significant changes (**Figures 2B, C**).

**Table 1:** Baseline characteristics of study cohort. Listed laboratory results were determined before transplantation.

Clinical parameter	LT patients	ACR	Non-ACR	p-value
	(n = 50)	(n= 12)	(n = 24)	
Age, years	50.6 (27–72)	50.3 (34–66)	50.8 (27–72)	0.9934
Sex				
Male/Female	32/18	9/3	17/7	>0.9999
BMI, kg/m^2^	26.5	26.3	26.6	0.7845
	(15.6−36.3)	(22.2−31.1)	(15.6–36.3)	
ACR grade				
Intermediate/mild	12	12	0	
Moderate/severe	0	0	0	
Primary disease				
HCC	7	3	4	0.6639
Alcoholic cirrhosis	7	3	4	0.6639
ALF	5	1	4	0.6457
PSC	2	0	2	0.5429
PBC	2	1	1	>0.9999
Cryptogenic cirrhosis	1	0	1	>0.9999
CCC	1	1	0	0.3333
ADPKD	1	0	1	>0.9999
NASH	1	0	1	>0.9999
Other	3	1	2	>0.9999
Retransplantation	6	2	4	>0.9999
**Recipient**				
labMELD	21.2	21.5	21	0.8298
	(6.5−40.8)	(6.5−40)	(8−40.8)	
INR	2.1	2.8	1.8	0.8214
	(1−7)	(1−7)	(1−5)	
Bilirubin total, mg/dl	11.5	12.1	11.1	0.9790
	(0.2−35.3)	(0.4−35.3)	(0.2−33)	
Creatinine, mg/dl	1.4	0.97	1.6	0.2487
	(0.5−7.56)	(0.5−1.8)	(0.5−7.56)	
**Graft**				
Surgical time, min	380.8	359.2	376.2	0.9929
	(189–898)	(297−487)	(189−898)	
Cold ischemia time, min	542.6	525.8	573.4	0.3309
	(67–901)	(314−673)	(67−901)	
Warm ischemia time, min	45.6	45.2	46.1	0.6971
	(30–68)	(34−62)	(31−68)	

HCC, Hepatocellular carcinoma; CCC, Cholangiocellular carcinoma; PSC, Primary sclerosing cholangitis; ADPKD, Autosomal dominant polycystic kidney disease; ALF, Acute liver failure; NASH, Nonalcoholic steatohepatitis; PBC, Primary biliary cholangitis; under other primary diseases Wilson disease and secondary biliary cirrhosis; are listed. Continuous values are presented as median values and categorical values as numbers. Continuous variables of ACR and non-ACR groups were assumed to be non-normally distributed and were tested using the Mann–Whitney U-test for pairwise analyses. Maxima and minima were given for continuous variables. Univariate differences between categorical variables were tested using the χ^2^ test or Fisher’s exact test.

**Table 2:** Baseline characteristics of the control group and LT patients.

Clinical parameter	Hernia patients (n = 19)	LT patients (n = 50)	p-value
*Age, years*	58 (29–81)	53 (23–72)	0.1358
*Sex*			
Male/Female	10/9	32/18	0.4189
*BMI, kg/m^2^*	27.4	26.5	0.2663
	(23–34)	(15.6–36.3)	

After discovering in the exploration group that EP increased following LT, we investigated the difference between patients who developed ACR and other graft dysfunctions to determine the possible relevance of EP in ACR. Patients who developed ACR had a significant increase in total, AnnV^+^, CD130^+^, Cx43^+^, MDR3^+^ and CD31^+^ EP (/µl) before surgery to POD 1(**Figure 2D**, p < 0.05). In contrast, ASGR1^+^ EP (/µl) did not differ between the ACR and non-ACR groups. Thus, elevated EP subpopulation levels in patients who later developed ACR demonstrated their potential utility to diagnose ACR.

### Discovering Single-Positive EP Profiles of ACR and Other Graft Dysfunctions

**Figure 3:**
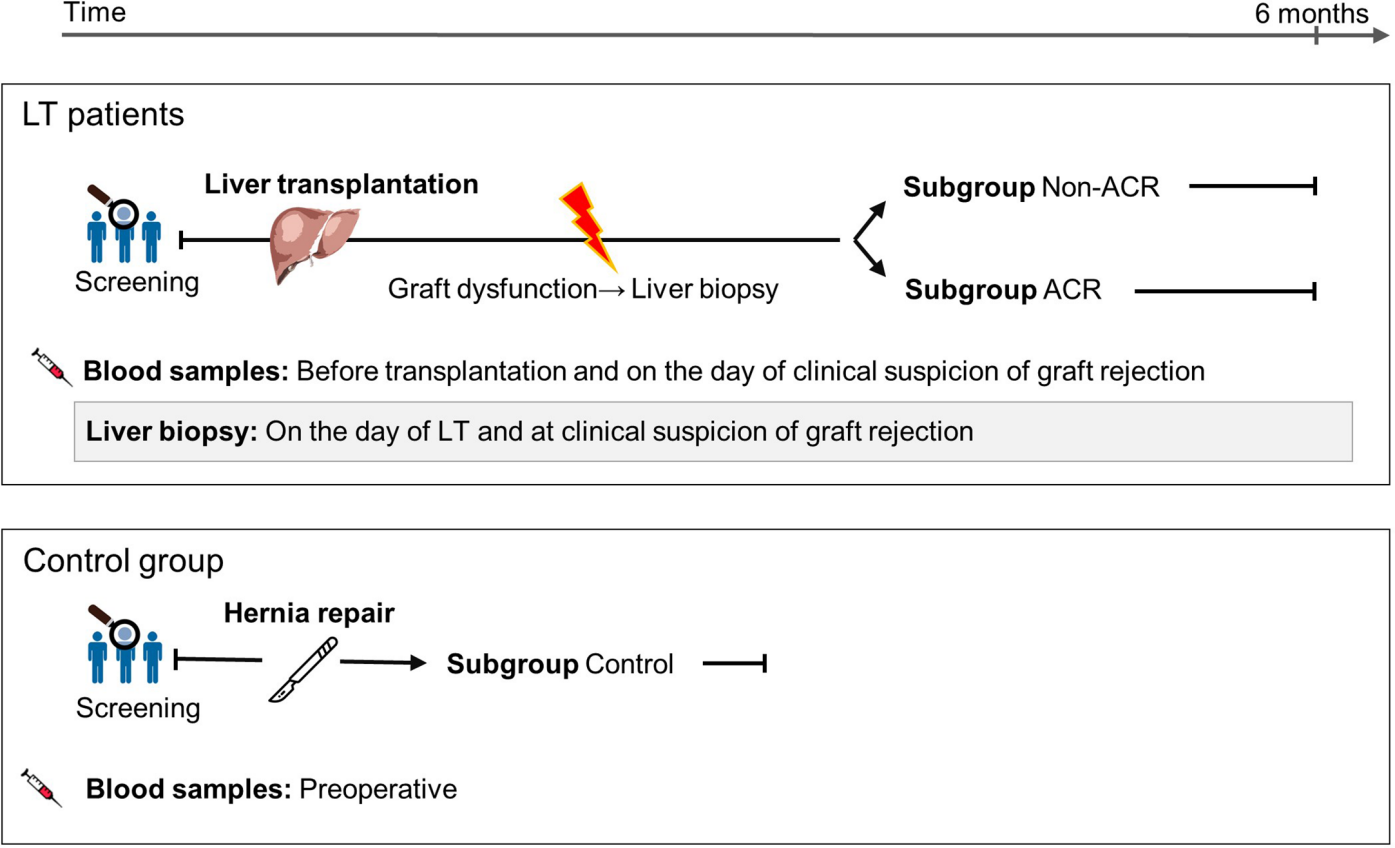
Study cohort description and patient sample classification for a EP diagnostic accuracy study on ACR following LT. Blood samples were obtained, accompanied by LBs due to suspicion of ACR based on abnormal liver parameters such as elevated bilirubin and transaminase levels or deterioration of synthetic liver function. Samples and patients were divided into ACR and non-ACR subgroups according to their biopsy result. The control group comprised patients with a symptomatic hernia, and their blood samples were collected before hernia surgery.

After observing in our exploration group that EP subpopulations were increased during the surgical stress response and that EP could provide an indication of ACR, we investigated EP as a diagnostic biomarker using a diagnostic accuracy study (**Figure 3**). As described in materials and methods, we stained EP with several antibodies. As a result, different EP profiles expressed a multitude of combinations of antigen positivity. In the following, we categorized those that had solely bound one antibody as single-positive EP and analyzed their profiles. In total, we analyzed the EP profiles of 95 blood samples, of which 76 were obtained with corresponding LBs due to suspicion of ACR based on abnormal liver parameters such as elevated bilirubin and transaminase levels or deterioration of synthetic liver function. In total, we obtained 19 biopsy-proven ACR samples and 57 biopsy-proven graft dysfunction samples with no sign of ACR (**Supplementary Figure 2**). The biopsy samples were analyzed according to the Banff classification of ACR, and 17 samples were verified as grade 1, 1 sample as grade 2 and 1 sample as borderline. In addition, we included a control group with no record of liver diseases (n = 19).

**Figure 4:**
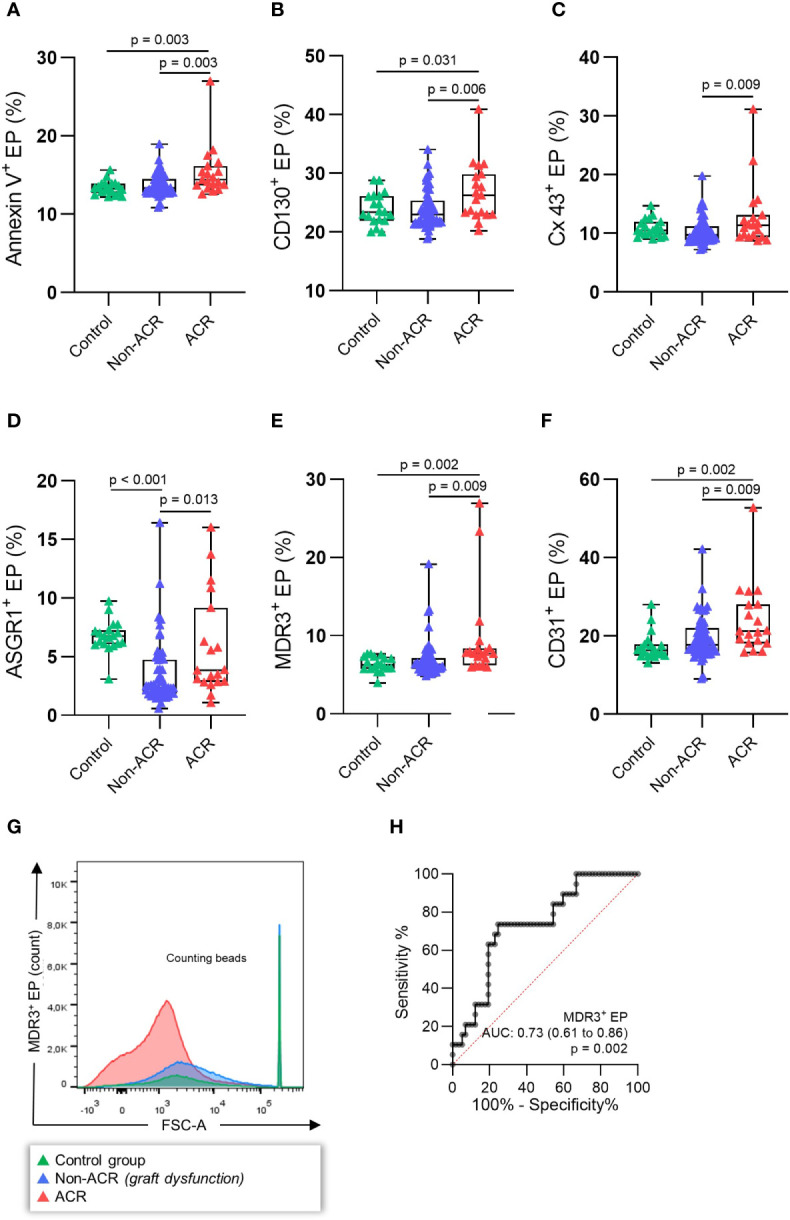
EP profiles during histology-proven ACR. EP were determined in the blood plasma of patients with concomitant LBs. The patients were then classified as histology-proven ACR or non-ACR. EP surface antigens **(A)** AnnV^+^, **(B)** CD130^+^, **(C)** Cx43^+^, **(D)** ASGR1^+^, **(E)** MDR3^+^, **(F)** CD31^+^ EP were stained, and relative EP (%) were analyzed. **(G)** Example FSC-A (forward scatter) histograms of MDR3^+^ EP (%) **(H)** demonstrating the highest receiver operating characteristic (ROC) of all single antigens. One-way analysis of variance followed by Tukey’s *post hoc* test was used. The plots are indicated by the median, and all error bars indicate the IQR.

This analysis revealed that AnnV^+^ EP (%) significantly increased in ACR samples compared to non-ACR samples as well as controls (**Figure 4A**, p = 0.003) The immunological (CD130^+^, Cx43^+^) and liver-derived (ASGR1^+^, MDR3^+^, CD31^+^) EP (%) also increased in ACR samples compared to non-ACR samples (**Figures 4B–F**, p < 0.05). ASGR1^+^ EP (%) increased in the control samples compared to non-ACR and ACR samples. The receiver operating characteristic (ROC) curves and area under the curve (AUC) for the respective EP subpopulations are shown in **Supplementary Figure 3**. Evidently, MDR3^+^ EP (%) exhibited the highest accuracy (**Figure 4H**, AUC: 0.73, 95% confidence interval [CI], 0.61–0.86), with a diagnostic sensitivity and specificity of 73.7% (95% CI, 51.2–88.2%) and 75.4% (95% CI, 62.9–84.77%), respectively. The cut-off value for distinguishing between ACR and non-ACR was >7.078% MDR3^+^ EP (%).

Taken together, there was a relative increase in immunological and liver-derived EP during ACR. MDR3^+^ EP (%) demonstrated the best diagnostic accuracy for distinguishing between ACR and non-ACR. All investigated surface proteins increased in the ACR samples compared to the control samples. In contrast, ASGR1^+^ EP (%) did not differ between the control and ACR samples, while non-ACR samples had lower EP levels than ACR samples.

### Modern Data Visualization Tools for Identifying Novel EP Populations

**Figure 5:**
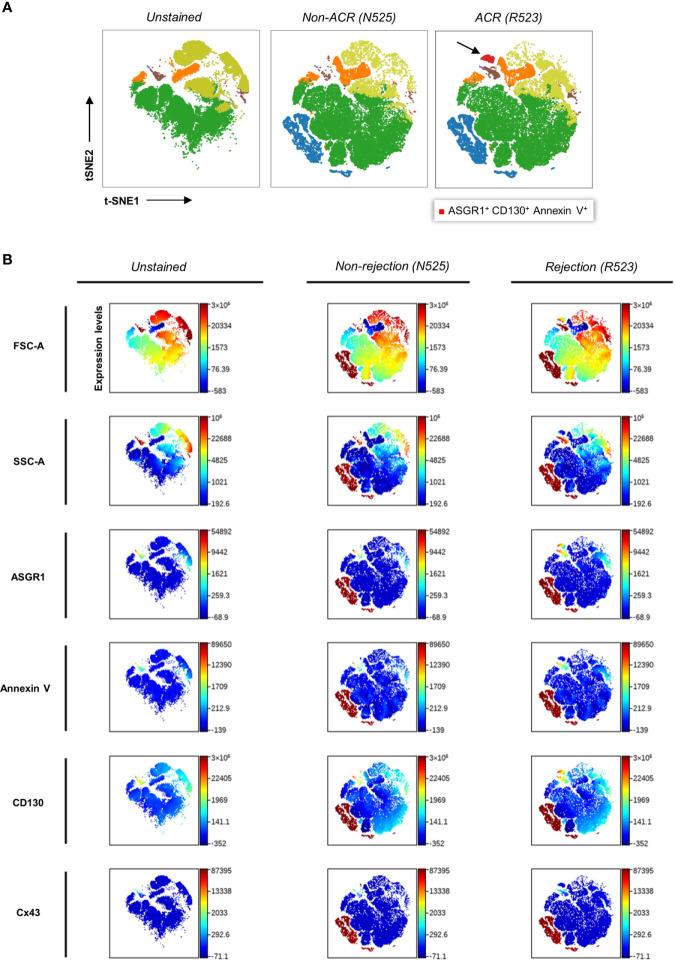
Clustering and color coding with FlowSOM and viSNE for identifying EP subpopulations *via* density plots. **(A)** Qualitative analysis and population identification were performed using viSNE and FlowSOM on samples of patient 52, who had episodes of graft dysfunction caused by ACR and non-ACR. Several LBs were performed over time, and their concomitant blood samples presented the following data. t-SNE (t-distributed stochastic neighbor embedding), an unsupervised nonlinear dimensionality reduction algorithm, was used to fit six-dimensional data into two dimensions. All clusters were created *via* FlowSOM analysis. Clusters were formed based on FACS channels. The arrow indicates the novel ASGR1^+^CD130^+^AnnV^+^ EP population. Coordinates for each t-SNE dimension (t-SNE1 and t-SNE2) were calculated for each microparticle after dimensionality reduction. **(B)** Color-coded t-SNE density plots showing different antigen expression levels by the channel of above-mentioned samples.

Further investigations were carried out to reveal the most sensitive combination of surface antigens that can distinguish ACR from non-ACR samples. The biaxial plots created from six cytometry channels from 76 patient samples yielded a data set suitable for dimensional reduction tools (**Supplementary Figure 4**). Therefore, we visualized multidimensional data using viSNE, an unsupervised nonlinear dimensionality reduction algorithm. This facilitated the search for novel EP populations and enabled the visualization of high-dimensional single-event data. After creating viSNE maps, we performed a manual search for discriminating populations. To enhance objective population discrimination, FlowSOM algorithms were additionally run, and EP metaclusters were formed depending on the antigen expression. ACR, non-ACR, and negative control samples were compared using these two algorithms, and the map representation differences between EP metaclusters were determined (**Figure 5A**). We identified an ASGR1^+^, CD130^+^, and AnnV^+^ EP subpopulation during ACR *via* viSNE density plots derived from different FACS channels (**Figure 5B**).

### Testing the Diagnostic Accuracy of the ASGR1^+^CD130^+^AnnV^+^ EP Population

**Figure 6:**
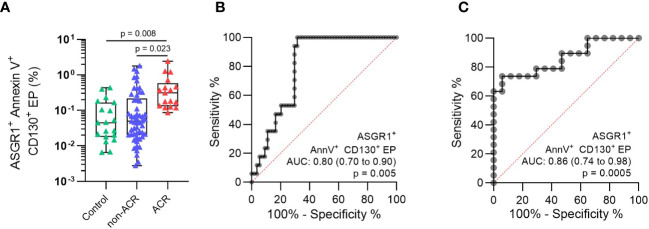
Analysis of ASGR1^+^CD130^+^AnnV^+^ EP (%) population discovered by viSNE maps and FlowSOM algorithm. **(A)** The particular EP subpopulation was identified in the plasma of patients with concomitant LBs. The patients were then classified as histology-proven ACR or non-ACR. ROC was constructed by comparing **(B)** ACR vs non-ACR and **(C)** ACR vs control. One-way analysis of variance followed by Tukey’s *post hoc* test was used. ROC curves and AUC were generated for relative EP. The plots are indicated by the median, and all error bars indicate the IQR.

The ASGR1^+^CD130^+^AnnV^+^ EP subpopulation was reevaluated in all samples relative to the total EP. These values were compared between the respective groups to determine its diagnostic significance and accuracy of differentiating ACR from non-ACR. This EP population increased significantly in the ACR group compared to the non-ACR (**Figure 6A**, p = 0.023). In addition, the values in the ACR group increased significantly compared to that of the control group (**Figure 6A**, p = 0.008). ASGR1^+^CD130^+^AnnV^+^ EP (%) exhibited the highest accuracy (AUC: 0.80, 95% CI, 0.70–0.90), with diagnostic sensitivity and specificity of 100% (95% CI, 81.67– 100.0%) and 68.5% (95% CI, 55.3–79.3%), respectively (**Figure 6B**). The cut-off value was >0.082% ASGR1^+^CD130^+^AnnV^+^ EP for distinguishing between ACR and non-ACR whose cross-tabulation was shown in **Table 3**. When the ACR and control groups were compared, the diagnostic accuracy was increased (AUC: 0.86; 95% CI, 0.74–0.98). The diagnostic sensitivity and specificity were 73.7% (95% CI, 51.2–88.2%) and 94.1% (95% CI, 73.0–99.7%), respectively (**Figure 6C**). In summary, this new EP population achieved the highest diagnostic accuracy for detecting ACR compared to the EP subpopulations with other surface antigen combinations.

**Table 3:** Diagnosis sensitivity and specificity of ASGR1^+^ Annexin V^+^ CD130^+ EP^ (%) for ACR in liver transplant.

ACR detection	No. of reference samples	
	*Truly positive*	*Truly negative*
	*(Histological assessment)*	*(Histological assessment)*
*ASGR1^+^ Annexin V^+^ CD130^+^ positive*	19 (TP)	17 (FP)
*ASGR1^+^ Annexin V^+^ CD130^+^ negative*	0 (FN)	37 (TN)

TP, true positive; FP, false positive; FN, false negative; TN, true negative.

## Discussion

The development of reliable non-invasive biomarkers with high sensitivity for ACR would undoubtedly improve postoperative care. Besides severe complications, LB is time-consuming and ties up specialized staff (4). Non-invasive biomarkers would not only facilitate an easier and less time-consuming diagnosis of ACR but also would consequently reduce morbidity and improve graft function of LT patients. Aiming for a non-invasive ACR diagnostic tool, we stained EP and analyzed their combinations with viSNE maps and FlowSOM algorithms. While detecting significant differences in single-positive EP profiles, we discovered a triple-positive EP subpopulation, termed ASGR1^+^CD130^+^AnnV^+^ EP. This novel EP subgroup exhibited the best test accuracy for distinguishing ACR from other graft dysfunctions and control samples. This approach has proven itself to be easy to operate and objective to analyze just in a couple of hours comparing to LB and its evaluation. To the best of our knowledge, this is the first study to examine EP in a diagnostic accuracy study for diagnosing ACR after LT (4).

First, we cultured human hepatocytes for *in vitro* experiments, and EP release was measured under TNF-α stimulation (**Figure 1**) since the aim of the experiment was to test whether EP are released under immunological stress. Widely known for its pleiotropic actions, TNF-α is released mostly by macrophages, T cells, natural killer (NK) cells, and neutrophils during alloantigen presentation and elevated during acute rejection with T cell parenchyma infiltration being a major histological criterion of ACR (27–29). In our experiments, the TNF-α stimulation stressed hepatocytes and increased the total amount of EP and specific EP subpopulations. We used a hepatocyte culture for our *in vitro* experiments in which MDR3 and CD31 have not been labeled. This well established protocol in our laboratory (22) allows hepatocytes to be selectively collected after centrifugation through Percoll, which eliminates other cells like endothelial cells and cholangiocytes. However, cholangiocytes and Kupffer cells, which also reside in the liver, are ACR-regulating cells along with endothelial cells and T cells. These cell-cell interactions were not considered in our *in vitro* model, although we could show for the first time that EP are released from hepatocytes after TNF-α stimulation, indicating that EP can be released from hepatocytes during immunological stress. At the same time, using innate immunity activating factors other than TNF-α in a co-culture could simulate the ACR-state more accurately and thus resulting in clinically more comparable EP profiles. For instance, a co-incubation of IL-6 and exosomes could be very useful for understanding the mechanisms of T cell proliferation in the ACR context in solid organ transplantation (15).

Next, we characterized the course of EP following LT more precisely in a small exploration subgroup (**Figure 2**). There was a postoperative EP increase until POD3, followed by a plateau phase. This is in line with cytokine profiles and immune cell populations following LT that may trigger EP release (30, 31). In addition, only ASGR1^+^ EP (/µl) decreased postoperatively, which may result from postoperative inflammation. ASGR1 is located on the sinusoidal, basolateral and less prominently on the lateral side of hepatocytes (32). In inflammation associated with liver cirrhosis, the receptor can shift towards the canalicular surface, with a corresponding decrease on the sinusoidal and lateral surfaces (33). This could be the reason for the inverse behavior of ASGR1^+^ EP (/µl) compared to the other EP populations. After assessing the temporal properties of EP, we examined whether the various surface markers can identify patients at risk for ACR. Although the sample size was small with n = 11, we could demonstrate that patients with ACR showed a higher relative increase from preoperatively to POD 1 compared to the control group (**Figure 2D**). Only ASGR1^+^ EP (/µl) tended to increase without statistical significance when ACR and non-ACR were compared.

Studies so far examined the ACR in kidney, heart, hand, and liver grafts. Qamri and colleagues investigated the role of endothelial CD31^+^CD42b^−^ EP in kidney and pancreas recipients (34). The analysis showed an increase in circulating endothelial EP associated with ACR, but not with non-ACR. Please note, their biopsy-secured sample size was relatively small (n = 14), which is why statistical comparisons showed no significance. Morel and colleagues investigated the contribution of endothelial cell activation on the release of procoagulant EP during ACR in cardiac recipients (35). Endomyocardial biopsy and blood sampling were performed on the same day in this study, and it was observed that E-selectin positive procoagulant EP were associated with ACR. Our group had been investigating the role of immunological EP (CD4^+^, CD8^+^, CD25^+^, CD31^+^, MHC) within the first week after transplantation (6). Although CD4^+^, CD8^+^, and CD31^+^ EP (%) were higher in the ACR group; blood samples were collected at several time points according to a protocol, independent from clinical ACR suspicion. It should be noted that index tests and reference tests have to match in time, which is overlooked in studies causing liquid biopsy to lose its meaning (4). Another case report tested the detection of donor-derived EP of a hand transplant recipient underwent ACR (36). In brief, endothelial and immune system-related EP have been investigated, whereas there are hardly any investigations regarding organ-specific EP, and certainly no studies evaluating diagnostic accuracy.

The concept of organ-specific EP as a liquid biopsy was applied in this present study for more precise statements about ACR diagnosis. Therefore, we arranged our index test sampling according to real-life clinical conditions. All LBs and concomitant blood sampling were performed on patients who showed graft dysfunction with ACR suspicion. Follow-up biopsy, as well as blood sampling, were not performed, which automatically excluded LT patients with normal graft functions. This meant that both ACR and non-ACR sample groups showed characteristics of graft dysfunction, which could not be specified further at the time of sampling. Since no follow-up biopsies were performed, which would have possibly included LT patients with normal graft functions, our control group including hernia repair patients with normal liver parameters provided us with a weaker comparison since they did not undergo LT.

In order to reach the absolute EP per µl plasma, the *equation 1* was used. In this way absolute EP numbers could be calculated in total and individually for each antigen. Since the blood samples originate from different patients showing considerable interpersonal variation, we calculated the respective relative value of each EP population to the total EP and reduced the variance while obtaining fewer outliers. Considering this, we first measured AnnV^+^ (%) subpopulations, since EP are most specifically labeled with PS ligands, and AnnV is the most popular EP labeling agent among PS ligands (7, 37). Interestingly, other studies demonstrated that significant proportions of EP are actually PS negative and thus Annexin V negative (38, 39). Annexin V^+^ EP were significantly increased in patients with ACR, when compared to non-ACR or controls possibly due to proinflammatory conditions promoting PS exposure (**Figure 4**). Therefore, we did not exclude Annexin V negative EPs from the analysis to avoid biasing the results between the experiment groups. Subsequently, we examined CD130^+^ EP (%) in more detail. Crucial to IL-6 engagement, CD130 is a transmembrane subunit important for signal transduction whose expression depends on cellular inflammation (40). The role of IL-6, not only as a potential biomarker (41) but also as a potential liver regeneration trigger after LT has been studied extensively in the context of graft rejection by various groups (42, 43). Furthermore, an increased expression of hepatic Cx43 might be associated with the severity of inflammation in cirrhosis and ACLF (acute-on-chronic liver failure) (17), and Cx43 knockout mice showed increased hepatocyte death, inflammation, and oxidative stress (44). We assumed that EP from hepatocytes should also carry Cx43 mediating a danger signal to other hepatocytes. This assumption was supported by our findings demonstrating increased Cx43^+^ EP (/µl) under TNF-α stimulation as well as during ACR. We concluded that this is a possible protective reaction of hepatocytes against the increased acute inflammatory environment. EP from resident cells, such as ASGR1^+^ EP, first emerged as a biomarker for distinguishing patients with liver malignancies from patients with cirrhosis but no malignancy (45). The fact that ASGR1 and MDR3 are specific to liver tissue and that their expression levels can change under inflammatory conditions led us to include them in our study showing significant increases in both EP populations during ACR.

Flow cytometry (FCM) is a well-established technique for high-throughput, quantitative, and multiparameter analysis of microscopic particles (8, 24, 45). The presented flow cytometric analysis was performed on a FACS BD LSRFortessa flow cytometer (BD Biosciences, Heidelberg, Germany). In a recent study by Welsh et al., they used the same flow cytometer among others and demonstrated for the first time that fluorescence and light scattering calibration of small particle data was suitable for standardized flow cytometry (46). They tested beads with a diameter from 152 up to 730nm and were able to detect them safely. Indeed, they demonstrated that the application of light scattering, fluorescence, and concentration calibration can result in highly consistent data between FCM platforms, independent of instrument collection angle, gain/voltage settings, and flow rate, providing a means for cross-comparison in standard units. To achieve the ultimate detection limits and finest calibration, it is crucial to use the suitable type of reference beads. In cellular vesicle detection, silica calibration beads are superior to polystyrene calibration beads since they exhibit forward scatter similar to that of cellular vesicles (47). For further qualification and single-particle analysis, other methods should be considered, such as TEM in our case.

Conventional cytometry data analysis, which displays only two dimensions simultaneously, ignores the complex structure and relationships between markers. Though viSNE plots resemble traditional biaxial plots, their efficiency comes from the simultaneous combination and representation of data from all dimensions. In this way, we sensitized cytometry plots to small subpopulations and facilitated the search for novel populations without the necessity of scanning through hundreds of biaxial plots. FlowSOM serves the same purpose by generating a SOM of clusters based on chosen markers and groups EP into populations. Utilizing viSNE maps and FlowSOM algorithms for qualitative data analysis and the detection of EP populations (48, 49), we could objectively identify and classify EP subpopulations with multiple target proteins robustly. Ultimately, we identified the ASGR1^+^CD130^+^AnnV^+^ EP population as a novel EP subpopulation, which could be used as a diagnostic biomarker for ACR following LT.

One of our limitations in this study is the exploratory characteristic of our report which respects most of the STARD criteria for reporting diagnostic accuracy study results. It is plausible in a next step to improve sensitivity/specificity of the novel revealed ASGR1^+^CD130^+^AnnexinV^+^ EP population to detect ACR. Furthermore, a validation study is needed to confirm our findings and increase the presented liquid biopsy method’s diagnostic robustness and collect longitudinal EP data from liver transplant patients without complications. Inclusion of other autoantigen panels to assess liver graft damage in the long term could reveal insight to autoimmune-mediated processes.

We have shown that hepatocytes release EP *in vitro* under stimulation by TNF-α, a cytokine involved in systemic inflammation which is also critical in ACR. Furthermore, levels of EP changed postoperatively after LT and increased when patients were at risk for ACR. Consequently, we recorded increased liver-derived and immunological EP equivalent to LB detecting ACR in a diagnostic accuracy study. Strikingly, viSNE maps and FlowSOM algorithms identified a novel EP subpopulation, namely ASGR1^+^CD130^+^AnnV^+^ EP (%), which exhibited the best test accuracy distinguishing ACR from non-ACR. Nevertheless, a validation study is needed to improve the accuracy of the ACR diagnosis and to transfer these findings to the clinic.

## Data Availability Statement

The raw data supporting the conclusions of this article will be made available by the authors, without undue reservation.

## Ethics Statement

The studies involving human participants were reviewed and approved by Ethikkommission der Charité Universitätsmedizin Berlin. The patients/participants provided their written informed consent to participate in this study.

## Author Contributions

KK, MS, CK, PB, KS, AL, and FK conceived and designed the analysis, collected the data, performed the analysis, wrote the paper. NB, K-KH, NR, LF, MF, JG, PL, WS, CB, and JP contributed data and analysis tools. All authors contributed to the article and approved the submitted version.

## Funding

This work was supported by grants from the German Research Foundation (DFG, SCHM2661/3-1 and SCHM2661/3-2) and the Federal Ministry of Economics and Technology (BMWI, F4245601SK6).

## Conflict of Interest

The authors declare that the research was conducted in the absence of any commercial or financial relationships that could be construed as a potential conflict of interest.

## Abbreviations

ACR, Acute Cellular Rejection; AnnV, Annexin V; ASGR1, Asialoglycoprotein Receptor 1; CD, Cluster of Differentiation; Cx43, Connexin 43; EP, Extracellular particles; FACS, Fluorescence-activated Cell Sorting; FlowSOM, Self-Organizing Maps; gp, Glycoprotein; IL, Interleukin; LB, Liver Biopsy; LT, Liver Transplantation; MDR3, Multidrug Resistance Protein 3; PS, Phosphatidylserine; viSNE, Visualization of T-Distributed Stochastic Neighbor Embedding.
