# Supplementary material for: Sensing Acute Cellular Rejection in Liver Transplant Patients Using Liver-Derived Extracellular Particles: A Prospective, Observational Study
Source: Front Immunol. 2021 May 5;12:647900. doi: 10.3389/fimmu.2021.647900 (PMC8131523; doi:10.3389/fimmu.2021.647900)
Supplement: Supplementary file 1 [file DataSheet_1.pdf]

Supplemental information

Fig. S1. The levels of in vitro TNF-α–induced human hepatocyte–derived absolute EP.

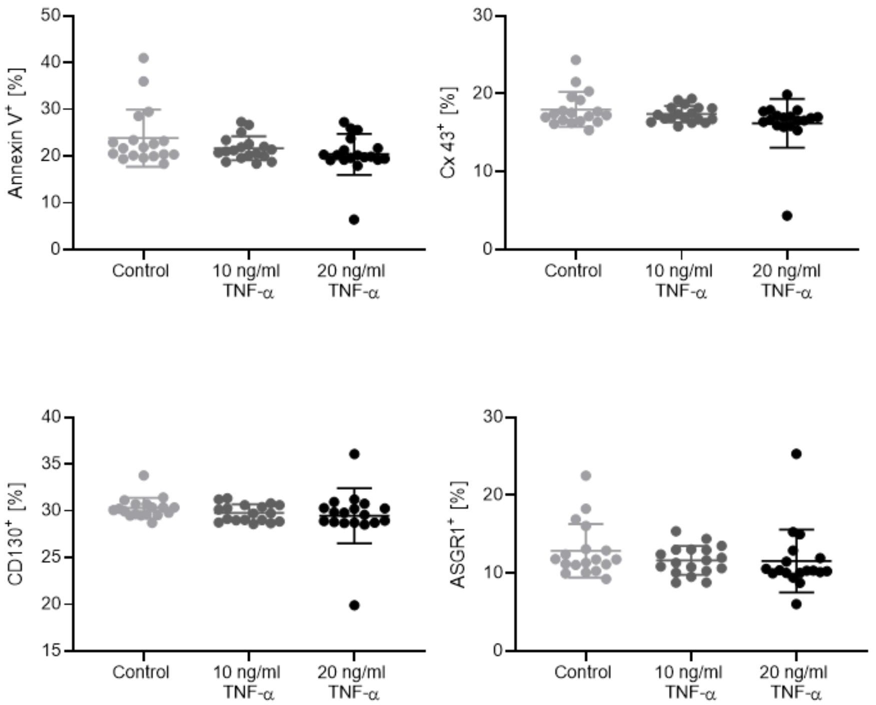

Primary human hepatocytes from liver resection were isolated, cultured overnight, and stimulated with 10 and 20 ng/ml TNF-α. $AnnV^{+}$, $CD130^{+}$, $Cx43^{+}$, $ASGR1^{+}$ EP were stained, and relative EP (%) were analyzed. The ordinary one-way ANOVA with Tukey's post hoc test was used. The plots are indicated by the median, and all error bars indicate the IQR.

Fig. S2. Recruitment process.

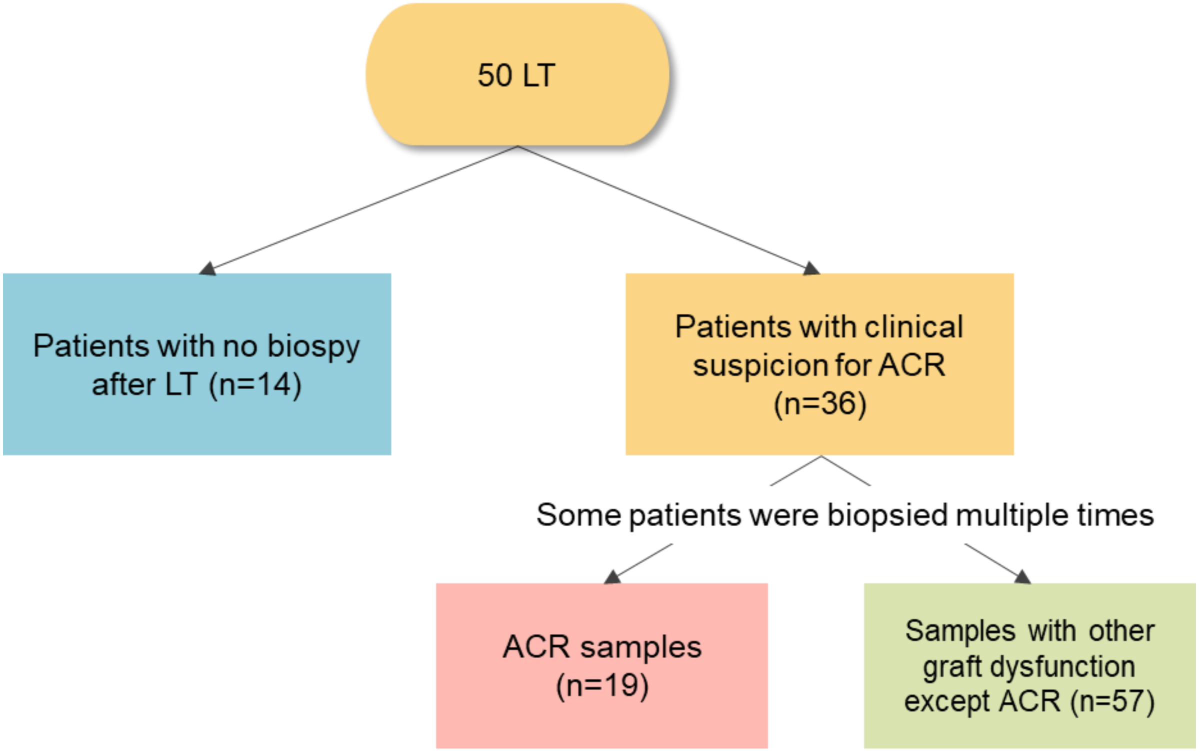

In total, 50 LT patients were recruited. After LT, 14 patients showed no clinical signs of graft dysfunction and left the hospital without complications. Thirty-six LT patients had clinical suspicion for ACR and were consequently biopsied. Due to multiple complications and graft dysfunction signs after LT, some patients were biopsied multiple times.

Fig. S3. ROC curves of EP antigens.

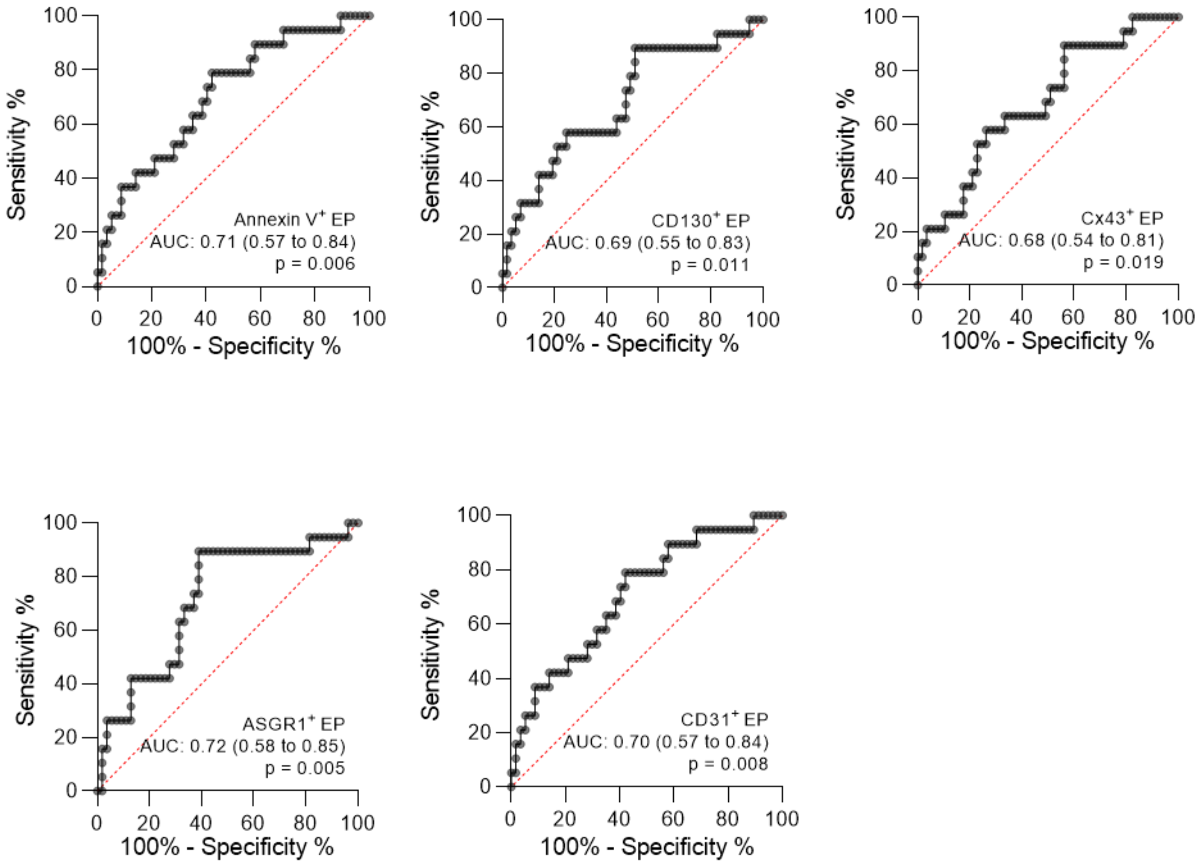

ROC curves of Ann V^{+}, CD130^{+}, Cx43^{+}, ASGR1^{+} and CD31$^{+\ EP}$ (%) were constructed by comparing ACR vs non-ACR.

Fig. S4. Reasoning behind using data reduction tools for EP flow cytometric data.

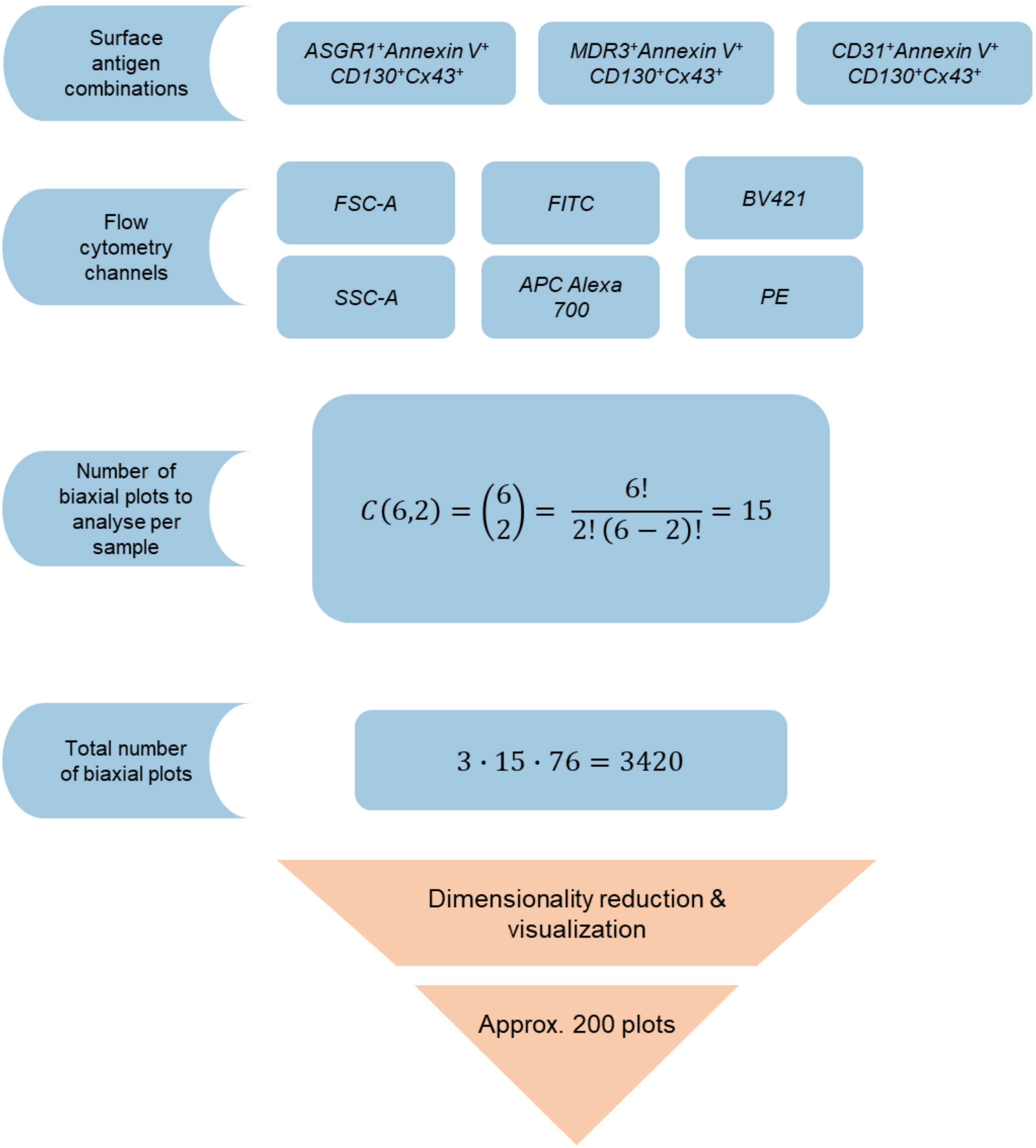

The number of biaxial plots for manual analysis was calculated as follows: the number of surface antigen combinations used in our measurements × combination of six flow cytometry channels × sample size. This could be reduced to approximately 200 biaxial plots to compare patient samples and discover new EP subpopulations.

Table S1. STARD 2015 guideline for reporting diagnostic accuracy in studies.

Section & Topic	No	Item	Reported on page #
**TITLE OR ABSTRACT**			
	**1**	Identification as a study of diagnostic accuracy using at least one measure of accuracy (such as sensitivity, specificity, predictive values, or AUC)	Abstract, p. 1
**ABSTRACT**			
	**2**	Structured summary of study design, methods, results, and conclusions (for specific guidance, see STARD for Abstracts)	No due to exploratory setting
**INTRODUCTION**			
	**3**	Scientific and clinical background, including the intended use and clinical role of the index test	Introduction, p. 2
	**4**	Study objectives and hypotheses	Introduction, p. 2
**METHODS**			
Study design	**5**	Whether data collection was planned before the index test and reference standard were performed (prospective study) or after (retrospective study)	Materials and Methods, p. 2
Participants	**6**	Eligibility criteria	Materials and Methods, p. 2
	**7**	On what basis potentially eligible participants were identified (such as symptoms, results from previous tests, inclusion in registry)	Materials and Methods, p. 2
	**8**	Where and when potentially eligible participants were identified (setting, location and dates)	Materials and Methods, p. 2
	**9**	Whether participants formed a consecutive, random or convenience series	Materials and Methods, p. 2
Test methods	**10a**	Index test, in sufficient detail to allow replication	Materials and Methods, p. 3
	**10b**	Reference standard, in sufficient detail to allow replication	Materials and Methods, p. 2
	**11**	Rationale for choosing the reference standard (if alternatives exist)	No alternatives for liver biopsy
	**12a**	Definition of and rationale for test positivity cut-offs or result categories of the index test, distinguishing pre-specified from exploratory	Materials and Methods, p. 3
	**12b**	Definition of and rationale for test positivity cut-offs or result categories of the reference standard, distinguishing pre-specified from exploratory	n/a
	**13a**	Whether clinical information and reference standard results were available to the performers/readers of the index test	Materials and Methods, p. 3
	**13b**	Whether clinical information and index test results were available to the assessors of the reference standard	Materials and Methods, p. 3
Analysis	**14**	Methods for estimating or comparing measures of diagnostic accuracy	Materials and Methods, p. 4

	**15**	How indeterminate index test or reference standard results were handled	n/a
	**16**	How missing data on the index test and reference standard were handled	Materials and Methods, p. 4
	**17**	Any analyses of variability in diagnostic accuracy, distinguishing pre-specified from exploratory	n/a
	**18**	Intended sample size and how it was determined	No due to exploratory setting
**RESULTS**			
Participants	**19**	Flow of participants, using a diagram	Suppl. Figure 2
	**20**	Baseline demographic and clinical characteristics of participants	Table 1 and 2
	**21a**	Distribution of severity of disease in those with the target condition	Results, p. 6
	**21b**	Distribution of alternative diagnoses in those without the target condition	n/a
	**22**	Time interval and any clinical interventions between index test and reference standard	Materials and Methods, p. 6
Test results	**23**	Cross tabulation of the index test results (or their distribution) by the results of the reference standard	Table 3
	**24**	Estimates of diagnostic accuracy and their precision (such as 95% confidence intervals)	Results, p. 8
	**25**	Any adverse events from performing the index test or the reference standard	None
**DISCUSSION**			
	**26**	Study limitations, including sources of potential bias, statistical uncertainty, and generalizability	Discussion, p. 12
	**27**	Implications for practice, including the intended use and clinical role of the index test	Discussion, p. 9,10
**OTHER INFORMATION**			
	**28**	Registration number and name of registry	DRKS00011631 /ICTRP Search Portal
	**29**	Where the full study protocol can be accessed	https://apps.who.int
	**30**	Sources of funding and other support; role of funders	Acknowledgment, p.13
